# Characteristics of Clear Cell Papillary Renal Cell Carcinoma (ccpRCC)

**DOI:** 10.3390/ijms23010151

**Published:** 2021-12-23

**Authors:** Jacek Rysz, Beata Franczyk, Janusz Ławiński, Anna Gluba-Brzózka

**Affiliations:** 1 Department of Nephrology, Hypertension and Family Medicine, Medical University of Lodz, 113 Zeromskiego Street, 90-549 Lodz, Poland; jacek.rysz@umed.lodz.pl (J.R.); beata.franczyk-skora@umed.lodz.pl (B.F.); 2 Department of Urology, Institute of Medical Sciences, Medical College of Rzeszow University, 35-055 Rzeszow, Poland; janlaw@wp.pl

**Keywords:** clear cell papillary renal cell carcinoma, clear cell renal cell carcinoma, papillary renal cell carcinoma, diagnosis, clinical features

## Abstract

Renal cell carcinomas (RCCs) is a group of various malignant tumours of the renal cortex displaying distinct clinical, morphologic, and genetic features. Clear cell papillary renal cell carcinoma (ccpRCC), belonging to this group, shares morphologic features with both clear cell renal cell carcinoma (ccRCC) and papillary renal cell carcinoma (pRCC) and therefore, more strict diagnostic criteria should be developed to avoid misdiagnosis. Despite overlapping features, ccpRCC has also distinct clinical behaviour, histologic characteristics (morphologic and immunohistochemical), and genomic features. The concepts concerning this tumour are constantly developing since its biological potential and molecular basis remains to be fully unravelled. First reports indicated the presence of ccpRCC in end-stage renal disease, and they underlined the enriched development in this group of patients; however, currently, it is known that such tumours can also occur spontaneously in the normal kidney. Numerous studies have demonstrated that clinical outcomes and prognosis of ccpRCC patients is highly favourable. Till now, no convincing evidence of metastatic ccpRCC or death caused by the disease has been found. Therefore, it is of high importance to correctly differentiate ccpRCC from other subtypes of RCC with a much worse prognosis and to introduce appropriate management.

## 1. Introduction

Renal cell carcinomas (RCCs) is a group of various malignant tumours of the renal cortex displaying distinct clinical, morphologic, and genetic features [1]. Clear cell papillary renal cell carcinoma (ccpRCC), belonging to this group, shares morphologic features with both clear cell renal cell carcinoma (ccRCC) and papillary renal cell carcinoma (pRCC) and therefore, more strict diagnostic criteria should be developed to avoid misdiagnosis [1,2]. Despite overlapping features, ccpRCC has also distinct clinical behaviour, histologic characteristics (morphologic and immunohistochemical), and genomic features due to which it was recognised by the World Health Organisation in 2016 as a new tumour entity [3]. In turn, the International Society of Urological Pathology (ISUP) Vancouver Classification of renal neoplasia uses the diagnostic term “clear cell (tubulo) papillary renal cell carcinoma (ccpRCC)” [4,5]. The concepts concerning this tumour are constantly developing since its biological potential and molecular basis remain to be fully unravelled. The prevalence of ccpRCC is estimated to range from 1 to 4.3%; this is the fourth most common subtype of renal cell carcinoma, preceded only by clear cell, papillary, and chromophobe renal cell carcinomas [6,7]. This type of tumour occurs generally at the age of 18–88 years with a mean of 70 years, and there is no gender predilection [4,5]. First reports indicated the presence of ccpRCC in end-stage renal disease (ESRD), and they underlined the enriched development in this group of patients; however, currently, it is known that such tumours can also occur spontaneously in the normal kidney and even a greater their prevalence has been described in non-end-stage renal disease patients [6,8]. Moreover, a great number of cases have been described in normal functioning kidneys as well as in conjunction with other renal neoplasms [9,10]. One of the reports suggests that this type of tumour may favour patients of African descent [11]. Currently, the causes of this disease are unknown since no specific genetic susceptibility for this entity has been identified yet [8]. Most studies have indicated its non-aggressive clinical behaviour. Therefore, some scientists even postulate the reclassification of this entity as a benign or low malignant potential neoplasm due to its aforementioned non-aggressive nature and the resemblance to several extrarenal benign neoplasms [12,13].

## 2. Macroscopic Findings

Clear cell papillary renal cell carcinoma can be solid or cystic, occasionally with flattened peripheral cysts [14]. It has been found that in general ccpRCC are small and well encapsulated (in a well-defined fibrous capsule) mix of branched glandular structures, cystic, papillary, tubular/acinar structures, solid sheet-like or nested components closely imitating clear cell renal cell carcinoma, all submerged in clear cytoplasm [6,8,15]. According to reports, these tumours are usually single and unilateral, however, some cases of multifocality and bilaterality were also described [16,17,18]. The cut surface of the ccpRCC tumour was found to be of tan-white, pink-tan, yellow, or red-brown colour [4,6,8]. No signs of necrosis were observed. In some cases, a focal haemorrhage is visible [19].

## 3. Microscopic Findings

Papillary formations of ccpRCC range from small blunt papillae in cystic spaces up to more complex branching structures with interconnecting ribbons resembling a hand with fingers [6,8,15]. They contain tightly packed secondary branching which gives the impression of a solid component [1]. Papillary architecture is not always prominent in clear-cell papillary RCC, which makes the diagnosis more difficult [16]. In cases with visible papillary architecture, the fibrovascular cores are usually thin and lined by a single layer of cells with the characteristic nuclear arrangement [16]. Sometimes, cystic changes are the predominant growth pattern in ccpRCC; such formations may be suggestive of multilocular cystic clear-cell RCC, however, specific nuclear arrangements accompanied by at least focal papillary architecture are indicative of ccpRCC. The size of cuboidal cells covering the papillae is usually small to medium; they comprise uniform nuclei facing the luminal surface [15]. Low-grade nuclei are arranged in the horizontal line apically distant from the basal membrane (except for the basement membrane) [5,20,21]. The nuclear atypia was found to correspond to Fuhrman Grade 1 or 2 [14,22]. Moreover, foci of large cells or eosinophilic cells can be also observed [21]. The lumina of the tubules or acini frequently contains proteinaceous secretion, while cystic spaces—seroanguinous fluid or colloid-like section [22]. Frequently, the stroma of ccpRCC is fibrous and displays smooth muscle metaplasia connecting with the tumour capsule [16,19,21]. What is interesting, the ccpRCC lack mitoses, pleomorphism, and hyaline globules [9]. Generally, in case of this type of tumour, the aggregation of foamy macrophages, hemosiderin depositions, psammoma bodies has not been observed. Additionally, lymphovascular invasion, renal sinus invasion, and tumour necrosis have not been reported to date [23]. Moreover, this type of tumour is lacking extrarenal extension, including vein or vein branch invasion [8]. The analysis with the use of electron microscopy revealed abundant glycogen with scant organelles in clear cell papillary renal cell carcinoma, while other studies indicated a lipid-depleted metabolic phenotype [24,25]. 

CcpRCC tumours display higher multifocality and bilaterality rates compared to ccRCC tumours [21,26]. The finding that the rate of multifocal and/or bilateral presentation in patients with ccpRCC is higher than that described for papillary or clear cell RCC may suggest the potential for either a hereditary or environmental component to these tumours [2]. Weng et al. [27] found that multifocal ccpRCC showed low histologic concordance (44%); the rate was lower than that for other multifocal RCC subtypes (59%) [28]. Due to the relatively high risk of histologic discordance in multifocal ccpRCC tumours, the diagnosis based on a biopsy sample collected only from a single tumour site does not preclude the presence of additional foci in the same kidney harbouring tumour with more aggressive RCC subtypes. Indeed, Weng et al. [27] described a case of one individual with multifocal ccpRCC and concomitant HG ccRCC in the same kidney which developed distant metastasis. Moreover, they suggested the existence of two types of multifocal ccpRCCs: “pure” and “mixed”, which differ phenotypically. They found that the risk of development of a different RCC subtype in the kidney of patients with “pure multifocal” ccpRCC in one kidney was reduced; however, these findings still require confirmation in larger cohorts. Table 1 presents macroscopic amd microscopic features of ccpRCC. 

## 4. Immunohistochemistry

Some immunoreactivity of ccpRCC overlaps with both clear cell and papillary renal cell carcinomas, however, this tumour has also its distinctive features. Rohan et al. [16] suggested that clear-cell papillary renal cell carcinoma could be closely related (or represent the same disease) with other entities, such as renal angiomyoadenomatous tumour, renal cell carcinoma with prominent leiomyomatous proliferation, and clear-cell renal cell carcinoma with diffuse cytokeratin 7 immunoreactivity due to overlapping many morphological and immunochemical features. 

Immunohistochemical analysis of ccpRCC cells revealed robust, diffuse positivity for cytokeratin (CK) 7 and cytokeratin 34βE12 as well as a cup-like staining pattern for carbonic anhydrase IX (CA9) which means that the basal and lateral membranes of the cells become labelled but not the apical membrane [16]. Carbonic anhydrase IX (CA9) is one of the well-studied HIF targets [16]. These aforementioned features seem to be unique for this type of tumour [16,29]. Samples of ccpCCR display positive immunoreactivity for parafibromin which is a tumour suppressor protein encoded by HRPT2 and a promising molecular marker for diagnosing parathyroid carcinoma [30]. Cui et al. [31] confirmed diffuse and strong nuclear positivity for parafibromin. The majority of tumours were also found positive for cytoplasmic staining of CK7 and negative for RCC-Ma [31]. These features can be used in the differential diagnosis from clear cell renal cell carcinoma which displays a higher positivity rate for RCC-Ma but is usually negative for CK7 and parafibromin, as well as from papillary renal cell carcinomas, which are typically positive for CK7 and RCC-Ma but negative for parafibromin [31]. Martignoni et al. [23] suggested that immunohistochemical expression of CK7 might not be sufficient to identify ccpRCC. They discovered that nearly all (92%) ccpRCC tumours were positive for 34βE12 [23]. Based on their results, they suggested that 34βE12 can be extremely useful for the recognition of this tumour, partly due to the presence of CK14 antigen expression. Moreover, ccpRCC was also found to be positive for vimentin, GLUT-1 and HIF-1, PAX2 and PAX8, CD133, cytoplasmic β-catenin, cytokeratin 19, E-cadherin, c-MET, and p27 [5,20,21,22,24,31]. HIF-1α and GLUT-1 proteins are markers of the activation of the HIF pathway [16]. However, also clear-cell RCC was demonstrated to be diffusely and strongly positive for GLUT-1 and HIF-1α. According to Rohan et al. [16], the overexpression of the aforementioned proteins may be associated with the inactivation of VHL in ccRCC. Leroy et al. [32] demonstrated also diffuse nuclear cyclin D1 immunoreactivity in most cases (83%). In turn, Williamson et al. [14] revealed that stroma was focally actin positive (94%), with sporadic desmin expression (13%). At the same time, this tumour was negative for α-methyacyl-CoA racemase (AMACR), cathepsin K, TFE3, TFEB, RCC Ma, renal cell carcinoma antigen, vinculin, oestrogen and progesterone receptor as well as parvalbumin. The labelling for CD10 is usually negative in solid areas, however, cyst lining areas could sometimes display minor focal labelling [14]. The positive staining for high molecular weight cytokeratin and GATA3 may imply a distal nephron phenotype, in spite of morphological similarity to clear cell renal cell carcinoma [23,33]. Finally, compared to renal cell carcinoma with smooth muscle or leiomyomatous stroma, the labelling for smooth muscle markers, including desmin or caldesmon, can be only poorly visible in ccpRCC pseudocapsule or even less in the stroma [34]. The results of immunohistochemical stainings are of key importance for the correct diagnosis of true ccpRCC. Deml et al. [9] used 25 various antibodies to characterise ccpRCC. They found significant differences in expression levels of parafibromin and hKIM-1 between ccpRCC/RAT and ccRCC/pRCC. Additionally, other studies demonstrated the potential of parafibromin to differentiate between ccpRCC and ccRCC and pRCC [35]. According to Deml et al. [9] also the CD70 can be a valuable marker in differentiating ccpRCC from ccRCC, due to the fact that its expression is unusual in ccpRCC and very frequent in ccRCC. In turn, Schwartz et al. [36] found that CD133 staining differentiates ccpRCC from other renal tumours. CD133 is a pentaspan membrane protein that may play a functional role as an “organiser” of plasma membranes. Additionally, another stem/progenitor cell marker OCT3/4 was observed to appear diffusely positive in ccpRCC. Two studies reported that about 33% of ccpRCC cases are positive for GATA3 [9,37]. This protein was suggested to be crucial for the regulation of the development as well as the function of Th2. Rohan et al. [16] demonstrated the expression of the high molecular weight CK—34βE12 in 56% of ccpRCC, but it was absent in the clear-cell or papillary RCCs. 

Immunohistochemical analysis can be useful in and differentiation between ccpRCC and ccpRCC-like tumours. Subsequent molecular analysis of chromosome 3p could support the ccpRCC diagnosis [23]. Williamson et al. [38] analysed the immunohistochemical profile of 14 ccpRCC-like tumours displaying high 3p deletion frequency (82%) that could not be distinguished from ccpRCC morphologically. They found that nearly all cases lacked the characteristic immunoprofile of sporadic ccpRCC, frequently presenting diffuse CD10 labelling (64%), negative or focal CK7 reactivity (55%), or both (18%). The prevalence of chromosome 3p deletion was comparable to that observed in clear cell renal cell carcinomas (80%). The authors concluded that sometimes patients with VHL disease might have a tumour that histologically resembled ccpRCC, however, they usually lacked the characteristic immunohistochemical and molecular profile [38]. Immunohistochemistry (but also molecular analyses) were suggested to be sometimes insufficient to distinguish ccpRCC from renal angiomyoadenomatous tumour (RAT) [9]. Table 2 presents immunohistochemistry markers in the described three types of rnal cancers

## 5. Molecular Pathways

Genetic studies of clear cell papillary renal cell carcinomas are sparse and probably therefore no disease-specific mutation has been identified to date. Several studies demonstrated various chromosomal aberrations in ccpRCC, including monosomy of chromosomes 16, 17, and 20 and trisomy of chromosomes 10 and 12 [13,18,39]. Characteristic genetic features that differ this tumour from clear cell and papillar renal cell carcinoma may include VHL gene mutations and 3p losses [9]. Some studies indicated the presence of VHL mutations in some ccpRCC cases, while other authors argue that the true ccpRCC cannot harbour abnormalities in the VHL gene [1,3,9,16,40,41,42]. The results of studies have indicated that normally ccpRCC lacks genetic alterations found in either clear cell RCC or papillary RCC—chromosome 3p deletion or polysomy of chromosomes 7 and 17 [4,22,43]. Additionally, the loss of chromosome Y is extremely rare in ccpRCC cases [18,22]. Compared to papillary RCC arising in ESRD, the incidence of gain of chromosome 16 or gain of chromosomes 7 and 17 in ccpRCC is greater or lower, respectively. ccpRCC and clear cell RCC also differ in the prevalence of copy number changes such as 5q gains and 8p losses [16]. Deml et al. [9] reported three chromosome 3p deletions in 20 ccpRCC (in 14.3% of cases). Similar 3p losses were described by Martignoni et al. [41], however, in their study, they were accompanied by VHL mutation. In turn, Shi et al. [24] revealed the presence of monosomy of chromosome 3 in some ccpRCC cases. It has been reported that only a small percentage of patients with von Hippel–Lindau (VHL) disease is affected by clear cell papillary renal cell carcinoma. Some authors even suggest that this diagnosis is uncertain as some of them may have tumours mimicking clear cell papillary renal cell carcinoma [38,44]. Despite initial results indicating the absence of VHL alterations in ccpRCC, the study of 27 cases performed by Deml et al. [9] demonstrated that the prevalence of VHL mutations was about 11%, while other groups found them even in 15–27% of cases [41,45]. However, still, the incidence of VHL gene alterations in ccpRCC is significantly lower than in ccRCC [9]. Discrepancies in the rate of mutations found in ccpRCC in many studies may be related to various methods of detection (such as single nucleotide polymorphism, genotyping array, Sanger sequencing), and differences in studied populations. The other thesis states that tumours with VHL alterations may represent ccRCC with morphology and immunoprofile closely mimicking that of ccpRCC and RAT tumours. Currently, the presence of VHL mutations/3p deletions is not utilised as a diagnostic feature of ccpRCC [9]. Instead, ccpRCC are diagnosed based on morphology and immunohistochemical profile (especially diffuse strong CK7 expression) [9]. 

VHL mRNA was found to be generally overexpressed in the ccpRCC tumour compared with non-neoplastic renal parenchyma and ccRCC cases carrying 3p losses and/or VHL mutations, despite the absence of VHL inactivating mutations or chromosome 3p losses [16]. This observation supports the thesis that the molecular mechanism underlying ccpRCC may be different from ccRCC. The product encoded by the VHL gene is involved in the regulation of transcription of several genes via the hypoxia-inducible factor (HIF) pathway [46]. The silencing of VHL expression was demonstrated to be associated with the overexpression of many proteins, i.e., HIF-1α, glucose transporter-1 (GLUT-1), as well as CA9 [47,48,49]. According to studies, the pathogenesis of ccpRCC may involve the activation of the HIF pathway. However, according to Rohan et al. [16], the HIF pathway in ccpRCC may be activated in a manner that is independent of VHL. Such a hypothesis was based on the finding of immunohistochemical co-expression of CA9, HIF-1, and GLUT-1 and the absence of VHL gene modifications [16,24]. HIF pathway activation may be associated with increased intra-tumoral sorbitol concentrations [50]. In turn, Fisher et al. [51] demonstrated the overexpression of ceruloplasmin (CP), and vimentin (VIM; 0.003, and <0.001, respectively) as well as relative overexpression of CP and nicotinamide N-methyltransferase (NNMT) (when analysed individually) in ccpRCC. Clear cell papillary renal cell carcinoma shows high levels of CP which is an acute-phase reactant protein overexpressed in many inflammatory situations, as well as in patients with renal cell carcinoma [52]. Moreover, the relative down-expression of AMACR, BAMBI (BMP and activin membrane-bound inhibitor homolog), and solute carrier family 34 (sodium phosphate) member 2 (SLC34A2) (*p* < 0.001, < 0.001, and = 0.014, respectively) were demonstrated in ccpRCC patients. The expression of AMACR, BAMBI, and SLC34A2 is low in clear cell papillary renal cell carcinoma. The absence of AMACR immunohistochemical staining is consistent with its low expression. However, no statistically significant differences in AMACR, BAMBI, SCHIP1 (schwannomin-interacting protein 1), SLC34A2, CA9, CP, or VIM expression were observed between clear cell papillary renal cell carcinoma and clear cell renal cell carcinoma [51]. CcpRCC is characterised by decreased NNMT levels in comparison to clear cell renal cell carcinoma. In turn, Fisher et al. [51] found increased VIM expression in clear cell papillary renal cell carcinoma. Vimentin is an intermediate filament participating in cell adhesion, migration, survival, and epithelial-to-mesenchymal transition [53] VIM was suggested to act as a renal cell carcinoma oncogene (regulated by miRNA-138) in clear cell renal cell carcinoma, however, its role in the pathogenesis of ccpRCC remains unclear and requires further examination [54]. Another analysis of 24 cases of a distinct low-grade renal tumour have confirmed diffuse, strong expression of cytokeratin 7 and vimentin [21]; it also demonstrated that CD10, racemase, RCC antigen, translocation factor E3, TFE3, and translocation factor EB were consistently negative. Subsequent comparative genomic hybridisation array study of seven cases failed to detect chromosomal imbalances. 

A recent study based on Next Generation Sequencing identified a non-synonymous T992I mutation in the MET proto-oncogene in ccpRCC [55]. Previous reports suggested the association of this gene with the development of hereditary pRCC [56]. The application of targeted next-generation sequencing (NGS) technology enabled the identification of several common tumour-associated mutation ‘hot-spots’ in ccpRCC [13]. Lawrie et al. [55] reported mutations within intronic regions close to splice junction (such as mutations in ERBB4, PTEN, and STK11 genes) which functional role remains unknown, synonymous mutations that do not alter the produced amino acid sequence, as well as non-synonymous mutations influencing the proto-oncogene, MET (the T992I and N375S mutational variants) involved in epithelial-to-mesenchymal transition (EMT). Raspollini et al. [57] investigated the nature of ccpRCC by examining mutations within the following genes: KRAS, NRAS, BRAF, PIK3CA, ALK, ERBB2, DDR2, MAP2K1, RET, and EGFR. No mutations in any of the investigated genes have been reported. This preliminary finding supports the thesis that ccpRCC might be indeed an indolent tumour. Additionally, the NGS analysis performed by Morlote et al. [1] identified 30 somatic, nonsynonymous variants in more than half (63.3%) of ccpRCC cases. The new variants, not previously reported in ccpRCC, were detected in the chromatin-remodelling gene—ASXL1 (p.D905V), ATM gene involved in the DNA damage response (p.C2464R), CDKN2A (p. R165S), EGFR (p.V765M), VHL (p.V74G, p.N78S, p.S80N), and ZRSR2 (p.R448_R449insSR). Other studies indicated that ASXL1 mutations were recently demonstrated also in pRCC associated with ESRD [58]. Based on the SIFT or Polyphen algorithms, 17 out of 30 identified variants were suggested to be deleterious or possibly/probably damaging, 33.3% were predicted to be neutral or tolerated, while in the case of 10% variants, the prediction score was not available. SNP analysis demonstrated copy number abnormalities and/or loss of heterozygosity (LOH) in 22.7% of the cases, including a gain of chromosomes 3, 12, and 18 as well as complex abnormalities in three other cases. 

Weng et al. [27] explored potential metabolic alterations in ccpRCC. They observed significantly lower mtDNA content in ccpRCC compared to other RCC subtypes. What is important, their study was limited to RCC tumours for which matched germline DNA was obtained from adjacent normal kidneys, to avoid bias related to differences in energetic demands between samples [25]. Additionally, other studies have demonstrated that ccpRCC displays severe depletion of mitochondrial DNA (mtDNA) which could imply the shift away from respiratory metabolism [25]. Based on metabolic or mitochondrial DNA assessment, ccpRCC has been suggested to have a more unique mechanism for respiration and energy processing [25,27]. 

**Figure 1:**
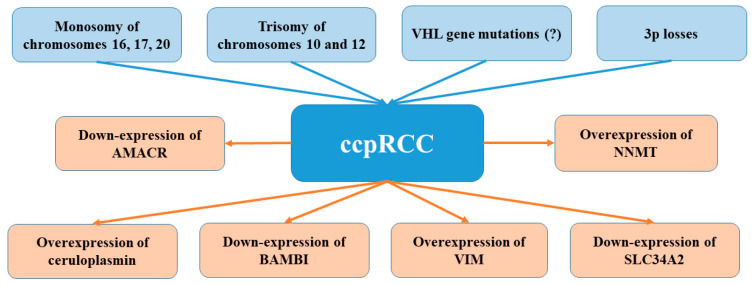
Presents molecular pathways involved in ccpRCC.

Apart from being metabolically distinct from clear cell RCC, ccpRCC has also a different pathological mechanism from both clear cell RCC and papillary RCC [55]. In turn, the study of non-coding RNA showed differences in expression of mature (entire miRnome), precursor (pre)-miRNAs, small nucleolar RNA (snoRNA), and small Cajal body-specific RNA (scaRNA) between ccpRCC and ccRCC or papillary RCC subtypes [55]. The analysis of non-coding RNA in ccpRCC demonstrated that the overexpression of all five members of the miR-200 family (miR- 200a, miR200b, miR200c, miR-141, and miR-429) was different from clear cell RCC and papillary RCC which showed low expression profile [55]. Lawrie et al. [55] suggested that enhanced expression of the above-mentioned miRNA could be involved in tumour suppression via the inhibition of epithelial–mesenchymal transition due to enhanced E-cadherin expression through direct targeting of ZEB1 and ZEB2 (encode transcriptional repressors of E-cadherin). Therefore, overexpression of the miR-200 family members may play a critical role in the repression of migration and invasion during cancer progression. Moreover, Deml et al. [9] have found immunoreactivity for E-cadherin and β-catenin in ccpRCC. All these findings may suggest the presence of incomplete or blocked epithelial-to-mesenchymal transition (EMT) in ccpRCC contributing to its indolent behaviour [55]. Indeed, until now, no studies have reported cancer-related death or metastasis in ccpRCC. Deml et al. [9] suggested that the indolent course of this tumour is associated with specific molecular alterations. Previously, they found that the expression of p27, CA-IX, CK7, and CK19 positively influenced the prognosis in sporadic RCC [59,60]. Additionally, ccpRCC samples displayed strongly positive staining for all of these markers, therefore it appears that such a specific signalling pathway may be partly responsible for the benign nature of these tumours. Recent studies have also revealed that ccpRCC lacks mutational drivers and has a characteristic degree of genomic instability displayed by other RCC subtypes [16,21,25,27,61]. Another study of miRNA profile revealed that 342 mature miRNA were differentially expressed in ccpRCC compared to normal samples with a false discovery rate (FDR) of less than 10% [62]. The comparison of three types of the tumour showed that the number of upregulated miRNAs in ccpRCC highly resembled those upregulated in ccRCC, while miRNAs downregulated in ccpRCC were similar to those downregulated in pRCC. Further analysis of miRNA profiles of ccpRCC, primary and metastatic ccRCC demonstrated that miRNA level of ccpRCC was more closely comparable with primary ccRCC than with metastatic ccRCC [62]. The highest differences in miRNA expression between ccpRCC and normal renal parenchyma were observed in case of miR-210, miR-122, miR-34a, miR-21, miR-34b*, and miR-489 (upregulated) as well as miR-4284, miR-1202, miR-135a, miR-1973, and miR-204 (downregulated). Shared miRNA patterns among ccpRCC and ccRCC could mirror an overlap in their underlying oncogenic mechanisms, including the hypoxia pathway. This pathway is associated with the expression of hypoxia-inducible factor (HIF)-related markers CA IX, glucose transporter 1, and HIF-1α. Additionally, Chang et al. [63] revealed that miR-210 expression positively regulated HIF and correlated with CA9 expression. The observation that miR-210 is upregulated in ccpRCC could mean that miR-210 can activate the hypoxia pathway even in the absence of VHL, however, this thesis requires confirmation. Munari et al. [62] also found that miR-122 was upregulated in ccpRCC tumours, however, its role in ccpRCC pathogenesis remains unknown. In ccRCC, this miRNA was suggested to be involved in the regulation of the VHL gene and was shown to be higher in primary compared with metastatic ccRCC [63,64,65]. Another miRNA overexpressed in ccpRCC, miR-34a, was previously indicated to suppress c-MYC and its complexes as well as to prevent cell invasion, thus acting as a tumour suppressor [66]. Again, the role of this miRNA in ccpRCC requires clarification. The upregulation of miR-18a was demonstrated in both ccpRCC and ccRCC compared to pRCC. Castellano et al. [67] demonstrated that the overexpression of miR-18a was involved in the negative feedback regulation of oestrogen receptor α (ERα) in breast cancer. In renal carcinoma cell lines, ERα serves as a proteasomal degradation target of the VHL protein [68], however, in the absence of VHL (e.g., ccpRCC) it does not undergo sequestration, but it promotes cell proliferation, enhancing the expression of miR-18a [62]. The expression of miR-135a and miR-204 was found to be downregulated in ccpRCC. According to studies, the first miRNA could act a tumour suppressor in RCC cell lines via the targeting c-MYC, while the second one is a VHL-regulated tumour suppressor acting through hindering macroautophagy [69,70]. However, the exact mechanism of these two miRNAs in ccpRCC merit further studies. It seems that ccpRCC is more similar to pRCC than ccRCC in terms of the profile of downregulated miRNAs. Figure 1 shows possible molecular pathways involved in ccpRCC.

## 6. Treatment and Management

Numerous studies have demonstrated that clinical outcomes and prognosis of ccpRCC patients is highly favourable. No recurrence or metastasis have been described in the case of clear cell papillary renal cell carcinoma with typical pathological features [8,14,22,26]. Even in cases of tumours presenting rare extrarenal extension, positive resection margin, necrosis, or intraoperative tumour disruption, adverse behaviour has not been reported [26,71,72]. Till now, no convincing evidence of death caused by the disease has been found [7,13,14,73]. Since clear cell papillary renal cell carcinoma seems to be of indolent nature, a nephron-sparing approach should be considered if possible [74]. However, its high multifocality and bilaterality rate (compared to ccRCC) complicates the management. Currently, the main treatments for renal cell carcinoma comprise radical nephrectomy or nephron-sparing surgery, including partial nephrectomy or thermal ablative techniques. Keeping in mind the tumour’s indolent biological behaviour, early detection of ccpRCC could help patients to avoid more invasive medical procedures [75]. According to estimations, half of the patients with this tumour are treated with nephrectomy, while the remaining individuals can be successfully managed through either cryo-ablation therapy alone or surveillance with serial imaging. The results of the study confirmed that nephrectomy-sparing measures are suitable in the management of ccpRCC patients, especially in those who are poor surgical candidates due to multifactorial circumstances. Current pieces of evidence indicate that none of the patients have had either metastatic disease or disease recurrence after nephrectomy [75].

## 7. Conclusions

Numerous studies have demonstrated that clinical outcomes and prognosis of ccpRCC patients is highly favourable. Till now, no convincing evidence of metastatic ccpRCC or death caused by the disease has been found [7,13,14,73]. Clear cell papillary renal cell carcinoma shares clinicopathological features with papillary renal cell carcinoma, however, the expression profile of eight studied genes was more similar to clear cell renal cell carcinoma [51]. Some more aggressive ccRCC tumours may mimic the features of ccpRCC, and therefore the establishment of distinct molecular profiles could enable definitive diagnosis in ambiguous cases. It is of high importance to correctly differentiate ccpRCC from other subtypes of RCC with a much worse prognosis and to introduce appropriate management.

## Figures and Tables

**Table 1:** The summary of macroscopic and microscopic features of ccpRCC.

Macroscopic Findings	Microscopic Findings
Solid or cystic tumour, rarely with flattened peripheral cysts	Tightly packed secondary branching resembling a solid component
Small and well encapsulated (well-defined fibrous capsule)	Not always visible papillary architecture
Mix of: - branched glandular structures, - cystic, papillary, tubular/acinar structures, - solid sheet-like or nested components closely imitating clear cell renal cell carcinoma	If papillary architecture is visible—the fibrovascular cores are usually thin and lined by a single layer of cells with the characteristic nuclear arrangement
All components submerged in clear cytoplasm	Cystic changes are sometimes predominant
Usually (but not always) single and unilateral	Cuboidal cells covering the papillae are usually small to medium
Tumour cut surface: - tan-white, pink-tan, yellow or red-brown colour - no signs of necrosis - sometimes focal haemorrhage	Low-grade nuclei lie in the horizontal line apically distant from the basal membrane (except for the basement membrane)
	The nuclear atypia was found to correspond to Fuhrman grade 1 or 2
	Frequent proteinaceous secretion in lumina of the tubules or acini Seroanguinous fluid or colloid-like section in cystic spaces
	Fibrous stroma displaying smooth muscle metaplasia
	Lack of mitoses, pleomorphism, hyaline globules, foamy macrophages, hemosiderin depositions, psammoma bodies, lymphovascular invasion, renal sinus invasion and tumour necrosis
	Higher multifocality and bilaterality rates compared to ccRCC

**Table 2:** The comparison of immunohistochemistry markers between ccpRCC, ccRCC, and pRCC.

	Clear Cell Papillary Renal Cell Carcinoma	Clear Cell Renal Cell Carcinoma	Papillary Renal Cell Carcinomas
Cytokeratin (CK) 7	Robust, diffuse positivity immunoreactivity	Negative immunoreactivity	Positive immunoreactivity
Cytokeratin 34βE12	Robust, diffuse positivity immunoreactivity	-	-
Carbonic anhydrase IX (CA9)	Cup-like staining pattern	Diffusely and intensively stained with a box pattern	Positive immunoreactivity (in tips of papillae)
Parafibromin	Diffuse and strong nuclear positivity immunoreactivity	Negative immunoreactivity	Negative immunoreactivity
RCC-Ma	Negative immunoreactivity	High positivity rate	Positive immunoreactivity
Vimentin	Positive immunoreactivity	Positive staining (more common in high-grade areas)	Diffuse cytoplasmic staining
Alpha-methylacyl-CoA racemase (AMACR)	Negative immunoreactivity	Variably positive immunoreactivity	Diffusely and strongly positive immunoreactivity
CD10	Negative or focally positive in most cases	Sawtooth pattern along a scalloped luminal contour	Variably positive immunoreactivity
GLUT-1	Positive immunoreactivity	Diffusely and strongly positive immunoreactivity	Positive immunoreactivity
HIF-1	Positive immunoreactivity	Diffusely and strongly positive immunoreactivity	-

## Data Availability

Not applicable.
